# Structural brain dynamics across reading development: A longitudinal MRI study from kindergarten to grade 5

**DOI:** 10.1002/hbm.25560

**Published:** 2021-07-01

**Authors:** Thanh Van Phan, Diana Sima, Dirk Smeets, Pol Ghesquière, Jan Wouters, Maaike Vandermosten

**Affiliations:** ^1^ icometrix, Research and Development Leuven Belgium; ^2^ Experimental Oto‐rhino‐laryngology, Department Neurosciences KU Leuven Leuven Belgium; ^3^ Parenting and Special Education, Faculty of Psychology and Education Sciences KU Leuven Leuven Belgium

**Keywords:** brain, childhood, cortical development, dyslexia, longitudinal, MRI, primary school, reading

## Abstract

Primary education is the incubator for learning academic skills that help children to become a literate, communicative, and independent person. Over this learning period, nonlinear and regional changes in the brain occur, but how these changes relate to academic performance, such as reading ability, is still unclear. In the current study, we analyzed longitudinal T1 MRI data of 41 children in order to investigate typical cortical development during the early reading stage (end of kindergarten–end of grade 2) and advanced reading stage (end of grade 2–middle of grade 5), and to detect putative deviant trajectories in children with dyslexia. The structural brain change was quantified with a reliable measure that directly calculates the local morphological differences between brain images of two time points, while considering the global head growth. When applying this measure to investigate typical cortical development, we observed that left temporal and temporoparietal regions belonging to the reading network exhibited an increase during the early reading stage and stabilized during the advanced reading stage. This suggests that the natural plasticity window for reading is within the first years of primary school, hence earlier than the typical period for reading intervention. Concerning neurotrajectories in children with dyslexia compared to typical readers, we observed no differences in gray matter development of the left reading network, but we found different neurotrajectories in right IFG opercularis (during the early reading stage) and in right isthmus cingulate (during the advanced reading stage), which could reflect compensatory neural mechanisms.

## Graphical abstract

In this three‐time point longitudinal MRI study, we show that during the first 2 years of primary school gray matter regions of the reading network seem to mature while stabilizing during the later grades of primary school. Children with dyslexia show a deviant cortical development in regions outside of the reading network.

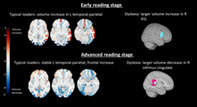

**Abbreviations**
DR: dyslexic readers
PBVC: percentage brain volume change
TR: typical readers

## 1 INTRODUCTION

Primary school years are very important for brain development since this time period is crucial for acquiring essential academic skills, such as reading and writing (Barquero, Davis, & Cutting, 2014). However, the number of longitudinal brain imaging studies during this period is limited and no information is available on cortical regional changes during specific stages of reading development. Therefore, it is still unclear how the cortical regions are shaped during primary school years and how their development would differ in children with learning difficulties, such as children affected by developmental dyslexia (i.e., severe and persistent difficulties with reading and/or writing). In addition, solid methodological approaches for reliably assessing structural changes from longitudinal pediatric data at the individual level are lacking. For this purpose, we applied a reliable dynamic brain measure adapted to pediatric data in order to investigate the cortical development during early and advanced stages of reading development in children with typical reading skills and children with dyslexia.

During primary school (from 5 to 12 years old), the brain undergoes structural changes that can be measured noninvasively using structural Magnetic Resonance Imaging (MRI). Over this period, the whole brain and the white matter volumes increase, while for the cortical gray matter, a plateau of the volume was observed during primary school (Lebel & Beaulieu, 2011; Mills et al., 2016; Sowell et al., 2003). Yet, although no substantial volume change seems to occur in the cortical gray matter at the tissue level from the beginning until the end of primary school, the volume locally increases or decreases at the sub‐region level. The volume peak has been shown to be reached the earliest in the primary sensorimotor and occipital visual areas and the latest in the higher‐order association areas (Aubert‐Broche et al., 2013; Brown & Jernigan, 2012; Gogtay & Thompson, 2010). As children rely on different learning strategies and competences over the different stages of primary school (Ullman, 2001), sets of brain regions might show a different cortical development in specific time periods, thereby paralleling the cognitive changes (Johnson, 2011). Groups of cortical regions that co‐develop (i.e., have similar neural trajectories) potentially represent brain networks (e.g., reading network; van Atteveldt, Vandermosten, Weeda, & Bonte, 2021). Hence, measuring cortical changes at specific periods of primary school would provide better insight in the development of brain networks involved in learning academic skills, such as reading.

Reading is an academic skill for which cortical development is important during primary school. In most adults, the reading network engages the inferior frontal, the temporo‐parietal and the occipito‐temporal areas (including the visual word form area; VWFA) in the left hemisphere (Pugh et al., 2001). However, this neural pattern is not innate and needs to be first formed via explicit teaching and practice. For example, prior to reading onset bilateral ventral occipital‐temporal regions are involved in processing visual categories, but once reading skills develop a neural specialization for processing words emerges in the left VWFA (Dehaene‐Lambertz, Monzalvo, & Dehaene, 2018). The change in functional neural pattern could imply that the brain structures also reorganize during this period. This reorganization is supported by studies showing changes in white matter structures when children learn to read (Vanderauwera, Wouters, Vandermosten, & Ghesquière, 2017; Wang et al., 2016) or when they undergo reading intervention (Huber, Donnelly, Rokem, & Yeatman, 2018). However, there is a lack of evidence for similar findings concerning the cortical gray matter, in particular during childhood. Structural differences in the cortical gray matter related to developmental dyslexia were frequently reported in areas involved in the reading network (Eckert et al., 2016; Linkersdörfer, Lonnemann, Lindberg, Hasselhorn, & Fiebach, 2012; Richlan, Kronbichler, & Wimmer, 2013). Those differences were suggested to be the neural cause of this neurodevelopmental disorder as they were also observed in prereading children and beginning readers (Beelen, Vanderauwera, Wouters, Vandermosten, & Ghesquière, 2019; Ozernov‐Palchik & Gaab, 2016). One study investigated structural gray matter differences in a longitudinal way between dyslexic and typical readers (TRs), and found that the cortical regions discriminating groups varied across primary school (Clark et al., 2014). Hence, those results indirectly suggest a divergent development of cortical regions in dyslexic readers (DRs) compared with TRs but neurodevelopment was not tested directly. For these reasons, quantifying the structural change rate during specific stages of reading would help in identifying potential atypical developmental patterns in children with dyslexia.

The small number of longitudinal studies investigating pediatric neuroanatomy is largely due to technical challenges in acquiring and analyzing pediatric MRI. Longitudinal designs are ideal to study brain development compared to cross‐sectional designs by better estimating and being more sensitive to brain changes. However, drawbacks in most of longitudinal pediatric studies are the sample size, in particular for children with neurodevelopmental disorders, and the use of nonadjusted neuroimaging processing methods for pediatric data, which leads to inaccurate estimation of structural measures (e.g., brain volumes; Phan, Smeets, Talcott, & Vandermosten, 2017). In addition, increased reliability of the structural MRI measures could be obtained when longitudinal T1 MRI data are integrated at the image level relative to the typical approach in which T1 data are processed independently for each time point and only integrated at the statistical level (e.g., via mixed effect models; Reuter, Schmansky, Rosas, & Fischl, 2012; Smeets et al., 2016; Smith et al., 2002). Those new longitudinal MRI methods are based on a within‐subject registration of images at different time points, and the linear and nonlinear transformations to align the images are then used to estimate the volumes. This enables to measure the change (i.e., difference of volumes) more directly and more reliably, and without any statistical assumptions regarding the model underlying the development (e.g., linear, quadratic, and cubic; Fjell et al., 2010). However, these longitudinal MRI methods have been implemented for adults and cannot directly be used in neurodevelopmental research as they assume a fixed head size over time, which is unlikely across primary school.

In the current study, we examined the cortical development throughout primary school and aimed to identify the potential atypical patterns related to developmental dyslexia. To this end, the volume changes in the cortical gray matter are directly measured at the image level using a within‐subject registration‐based method adapted to pediatric data, namely by considering head growth, resulting in a more reliable measure (see Methods). First, we used the proposed measure (PBVC_+scaling_) to examine the volume changes in total gray matter and across 62 cortical regions in children with typical reading skills. This enabled to determine how and which brain regions co‐develop during the early stage of reading (from the end of kindergarten to the end of grade 2) and during the advanced stage of reading (from end of grade 2 to the middle of grade 5). This might give an indication about which networks are formed during specific periods of primary school. Finally, we assessed in which of the 62 sub‐regions children with dyslexia have atypical developmental patterns compared to TRs during the early and advanced stage of reading.

## 2 MATERIALS AND METHODS

### 2.1 Pediatric MRI dataset description

The longitudinal data of children with dyslexia and with typical reading skills at three different time points in primary school come from the study of the Dyslexia Research Collaboration (DYSCO) project of KU Leuven. At baseline, 87 Dutch‐speaking children were recruited when they were in kindergarten, with half of the participants having a family risk for dyslexia and the other half without family risk. The family risk is defined as having at least one first‐degree relative with an official diagnosis of dyslexia (Vandermosten et al., 2015; Vanvooren, Poelmans, Hofmann, Ghesquière, & Wouters, 2014). Children were also assessed for the handedness based on the Edinburgh inventory (Oldfield, 1971). The following exclusion criteria were applied: low nonverbal IQ, history of brain damage, history of psychiatry disorders or visual deficits. The children at prereading stage did not yet receive formal reading and writing instruction in line with the guidelines of the Flemish government (http://www.ond.vlaanderen.be/). The children were then retrospectively classified as children with dyslexia or with typical reading skills based on literacy achievement at the third, fourth, and fifth grade of primary school, assessed with standardized word reading and pseudo‐word reading tests (Van den Bos, Spelberg, Scheepsma, & DeVries, 1994; Brus & Voeten, 1973; Dudal, 1997). Children were considered as having dyslexia when they had scores below percentile 10 on the same standardized reading test at all three time points. This strict classification of DRs incorporates the severity and persistence criteria defined in DSM‐V (American Psychiatric Association, 2013).

Out of the original sample, 75 children participated in the first MRI scanning session in which T1‐weighted were acquired at the end of the last year of kindergarten (TP1, age: 6.5 ± 0.5 years old). At the end of second grade of primary school (TP2, age: 8.2 ± 0.5 years old), 62 children of the same cohort were scanned again and at the middle fifth grade (TP3, age: 10.5 ± 0.5 years old), 53 children came again for a second or third acquisition. In order to measure the developmental change directly between two scans, we selected 41children for which at least one pair of time points (TP1–TP2 or TP2–TP3) of usable scans (i.e., not affected with severe head motion) was available. Based on the family risks and on the diagnosis for dyslexia, we categorized the children into two reading groups, resulting in 15 dyslexic children (DR) and 26 typical readers (TRs), both groups including children with family risk (11 DR and 13 TR) and without (4 DR and 13 TR). Demographics for the subjects per pair of time points are presented in Table 1.

**TABLE 1:** Demographics of subjects selected for the study over the pair of time points

Demographic	TP1–TP2	TP2–TP3
	Early reading stage	Advanced reading stage
Sample size	36	30
Age (years): mean (STD)	6.5 (0.5)–8.2 (0.4)	8.2 (0.4)–10.5 (0.4)
Handedness: mean (STD)	+0.7 (0.6)	+0.6 (0.6)
Gender: F/M	11/25	8/22
Family risk: with/without	23/13	15/15
Reading groups: DR/TR	13/23	10/20

*Note*: Children were scanned at three time point (TP) over primary schools: end of kindergarten (TP1), end of second grade (TP2), and middle of fifth grade (TP3).

The structural T1‐weighted images were acquired in a 3T MRI scanner (Philips, Best, the Netherlands) with a 32‐channel head coil, with the following parameters: 182 contiguous coronal slices, repetition time = 9.7 ms, echo time = 4.6 ms, flip angle = 8°, voxel size = 0.98 × 0.98 × 1.20 mm^3^, acquisition time = 6:22 min.

This study was approved by the Ethical Committee of the University Hospital of Leuven [B322201214607] and for all participants, informed consent was obtained from their parents according to the Declaration of Helsinki. The conditions of our ethics approval do not permit public archiving of anonymised study data, since consent had only been obtained for the participation in the study, and not to share data with third parties. Researchers seeking access to the study data should contact the last author (maaike.vandermosten@kuleuven.be) explaining the purpose of their request. In accordance with the EU general data protection regulation (GDPR), data will be released to requestors upon the following conditions: consent of the representative of the minor and a formal agreement between parties. Please note that the MRI data cannot be shared under any circumstance, as MRI data are person‐specific and therefore cannot be considered anonymous.

### 2.2 MRI processing pipeline for measuring cortical development

The MRI processing was performed with the cross‐sectional and longitudinal pipelines of icobrain version 5.0.1. (Struyfs et al., 2020) to analyze the longitudinal MRI of children. In the cross‐sectional pipeline of icobrain, the segmentation of the brain tissues and of 62 cortical gray matter regions was obtained per time point for each subject. The longitudinal pipeline enabled to obtain the brain volume change between two time points.

#### 2.2.1 Step 1: cross‐sectional pipeline for brain segmentation

**FIGURE 1:**
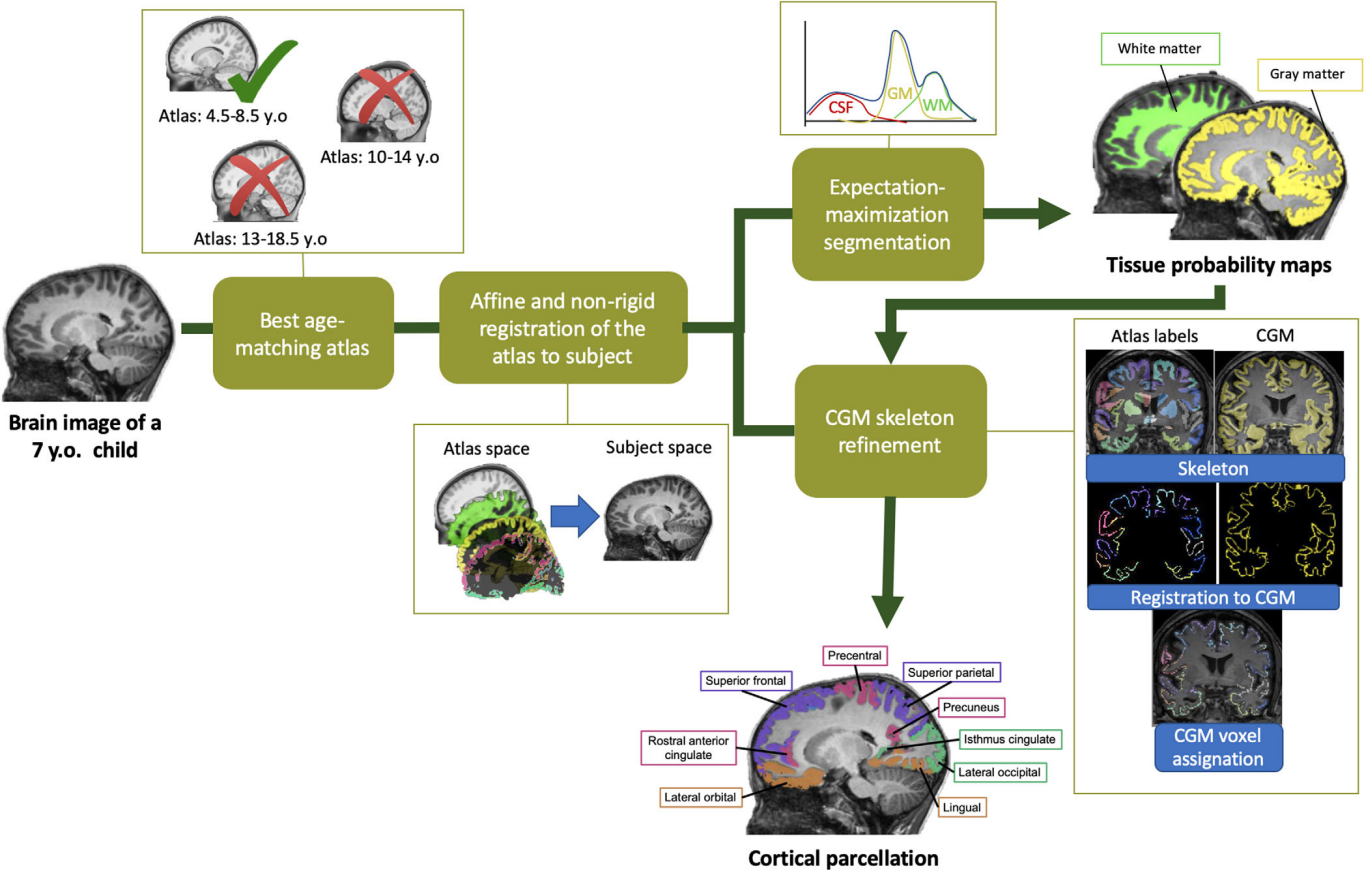
Main processing steps of the cross‐sectional pipeline of icobrain (v5.0.1) to obtain the segmentations of the brain tissues and the 62 cortical gray matter sub‐regions

In Figure 1, the cross‐sectional pipeline is depicted. In order to adapt the brain MRI processing method to pediatric populations, the first processing step is to select the best age‐matching atlas among independent pediatric population‐based atlases representing five age groups of the development (i.e., prepuberty, pre‐ to early puberty, pre‐ to mid puberty, early to advanced puberty, and post puberty), which are publicly available from the NIH‐funded MRI study of normal brain development (Evans, 2006; Fonov, Evans, McKinstry, Almli, & Collins, 2009). The best atlas is selected based on the subject age and the similarity between the subject brain and the atlas brain measured with the normalized mutual information. The age‐specific atlases consist of a head/brain template, a brain mask and probability maps of the three main tissues (i.e., gray matter, white matter, and cerebrospinal fluid). We also defined and adjusted the label maps of cortical regions from Mindboggle adult atlas (Klein & Tourville, 2012) on the age‐specific atlases with affine and nonrigid registration and manual corrections.

The whole brain segmentation is done similarly as described in our previous work (Phan et al., 2018). In summary, after skull‐stripping, bias correction and atlas to image registration, the probability maps of the three main tissues are obtained by the formulation and optimization of a Gaussian Mixture Model that considers the image intensities, the spatial prior knowledge of the tissues, the intensity nonuniformities caused by the bias field and the spatial consistency modeled by a Markov Random Field. From this step, total cortical gray matter is extracted from the gray matter probability maps.

The cortical gray matter is then parcellated into 62 regions based on the cortical gray matter probability maps computed previously and the parcellation map defined in the age‐specific atlas. Initial nonrigid registration between the subject T1‐weighted image and the atlas template is used to obtain a first propagation of the cortical labels from atlas space to subject T1‐weighted image space. This label propagation is further refined through a second nonrigid registration between the skeleton of the subject's binarized cortical gray matter segmentation and the skeleton of the binarized propagated atlas cortical labels. Finally, each cortical gray matter voxel is assigned to the cortical label, which matches the closest voxel in the skeleton of the nonrigidly propagated atlas cortical labels. Brain volumes of each cortical regions are computed as the sum of probabilities to be gray matter tissue in voxels assigned as the cortical region of interest multiplied by the voxel size. The 62 cortical gray matter regions are listed in the Table of Supporting Information II.

#### 2.2.2 Step 2: Longitudinal pipeline for measuring the percentage brain volume change (PBVC_+scaling_)

**FIGURE 2:**
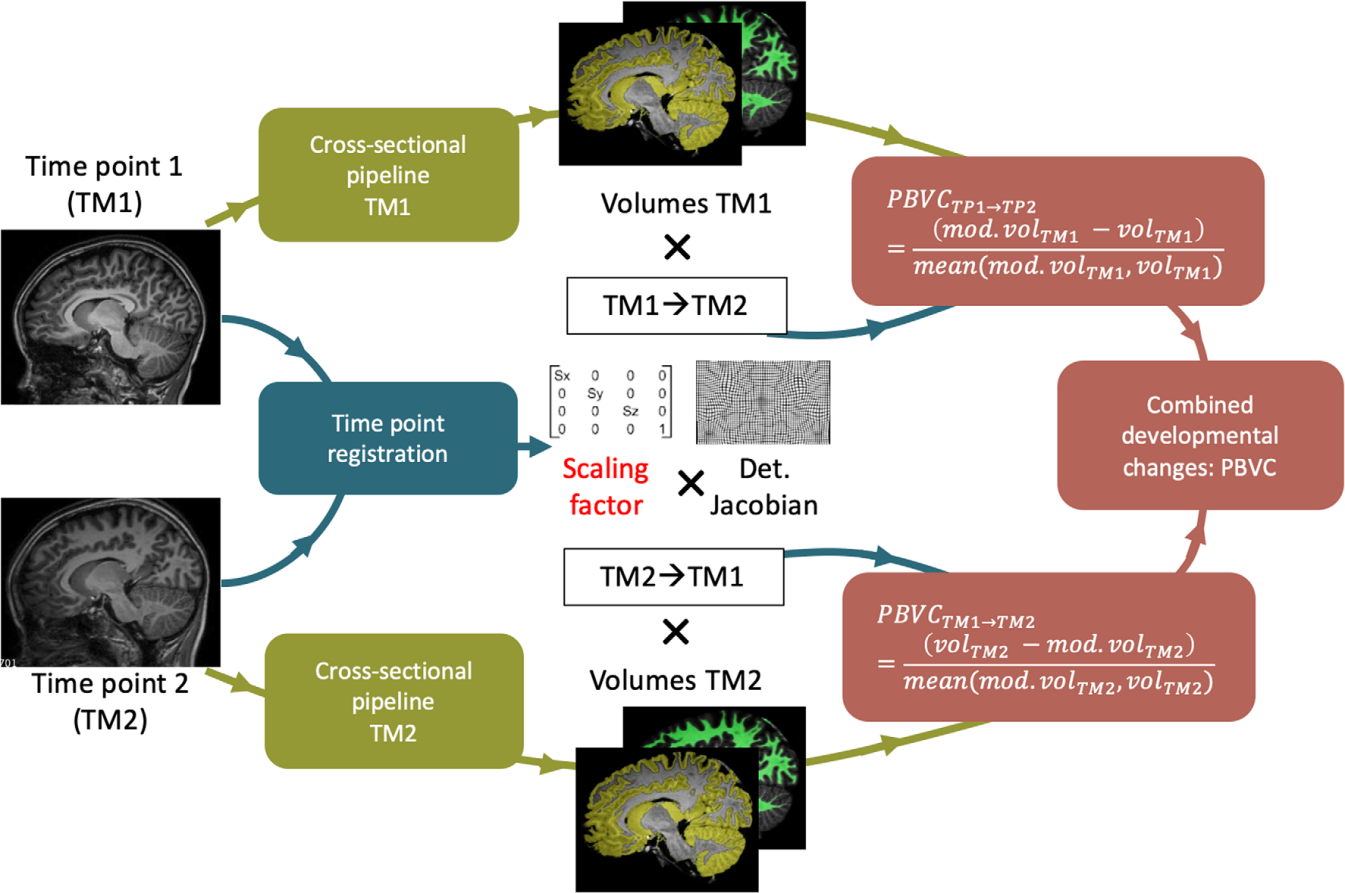
Main processing steps of the longitudinal pipeline of icobrain (v5.0.1) for computing the percent brain volume change (PBVC). The PBVC_+scaling_ results from the combination of the estimated PBVC, from the volume at time point 1 (TM1) and then from the volume at time point 2 (TM2), by the modulation of the scaling factor extracted from the affine registration of the head images and the Jacobian determinant of the deformation field obtained from the nonrigid registration of the brain images

The percent brain volume change (PBVC) corresponds to the difference of volumes between two time points for the same individual. It is defined as follows:

$$\text{PBVC}\;\left[\%\right]=100*\frac{{\mathrm{vol}}_{\mathrm{TM}2}-{\mathrm{vol}}_{\mathrm{TM}1}}{\text{mean}\left({\mathrm{vol}}_{\mathrm{TM}2},{\mathrm{vol}}_{\mathrm{TM}1}\right)}$$

where ${\mathrm{vol}}_{\mathrm{TM}1}$ is the volume of a brain structure at the first time point, and ${\mathrm{vol}}_{\mathrm{TM}2}$ is the volume at the second time point. The main processing steps in order to compute the PBVC are the same as applied for measuring the brain atrophy in adults with multiple sclerosis at the individual level (Smeets et al., 2016), but adapted to pediatric populations by considering the head growth (see Figure 2). Based on pairs of T1‐weighted images, the brain volume change is computed directly at the image level and not simply from the difference of brain volumes obtained independently at each time point (i.e., cross‐sectional method). To do so, the head images at two different time points are affinely registered and the brain images are then nonrigidly registered from the baseline time point to the other time point (i.e., time point registration) using NiftyReg (Modat et al., 2010; Ourselin, Roche, Prima, & Ayache, 2000). The default parameters were used for the affine registration and the parameters for the nonrigid registration were set as follows: number of levels = 1, bending energy penalty = 0, grid space = 2.5 mm, Jacobian determinant penalty = 0.01, maximum iteration per level = 40. The volume at the other time point (${\mathrm{vol}}_{\mathrm{TM}2}$) is then estimated from the volume at the baseline time point (${\mathrm{vol}}_{\mathrm{TM}1}$ **)** modulated by the scaling factor of the affine matrix (for considering the head growth) and the determinant of the Jacobian of the deformation field (for considering the local structural changes) obtained from the time point registration. The brain volume change in one direction (from the baseline to the other time point) is then defined as the difference between the modulated volume and the volume at the baseline time point divided by the average of both volumes. In the next step, the longitudinal change is also computed in the opposite direction (from the other time point to the baseline). Finally, the PBVC between the two time points corresponds to the average of the longitudinal changes in both directions. Our main adaptation to pediatric data is the modulation of volume with the scaling factor, enabling the measure to also consider the changes occurring due to head growth: PBVC_+scaling_. In the main text, we report on PBVC_+scaling_ of total cortical gray matter and PBVC_+scaling_ for each of the 62 cortical gray matter regions. In Supporting Information II, we also provide the PBVC of total white matter, deep gray matter, and whole brain volume.

#### 2.2.3 Validation of the proposed method

Given that the proposed measure (PBVC_+scaling_) had never been applied to developmental data, we examined the necessity to take head size growth into account in primary school children and we tested the test–retest reliability relative to a cross‐sectional measure. To this end, we used‐ besides the DYSCO‐dataset‐ the test–retest pediatric samples of Nathan Kline Institutes (NKI), a publicly available dataset (Zuo et al., 2014), which contains test–retest samples of anatomical (T1), functional and diffusion MRI acquired on healthy children from 6 to 17 years old. Out of the whole dataset, we selected 70 subjects for whom test–retest T1‐weighted images were available.

**FIGURE 3:**
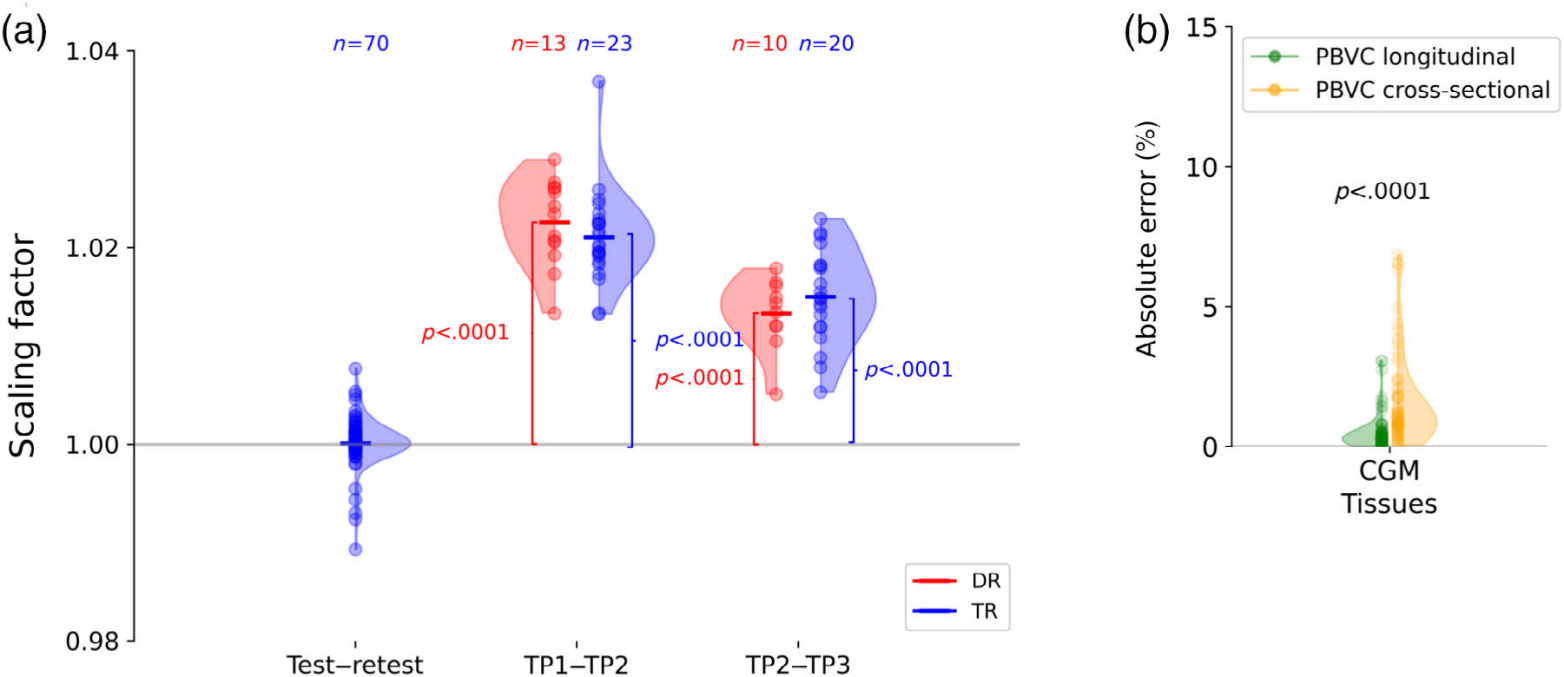
The proposed method (i.e., longitudinal PBVC_+scaling_), which considers head growth, is relevant and reliable for assessing cortical development during the early and advanced stages of reading. (a) The scaling factor is not significantly different from 1 for the test–retest data (DKI‐sample) but is different from 1 for the DYSCO‐sample during both the early (TP1–TP2) and advanced (TP2–TP3) stage of reading, indicating substantial head growth for each reading stage. (b) Estimating the PBVC_+scaling_ with the longitudinal method leads to a smaller error rates compared to the cross‐sectional method

First, we examined whether adjusting for the head growth was relevant for investigating brain changes at the different stages of reading during primary school. The head growth was measured with a scaling factor retrieved from the affine registration of the head images at two different time points. With a one sample *T*‐test, we tested the null hypothesis that the scaling factor (as a measure of the head growth) is equal to one (corresponding to no change in size). As illustrated in Figure 3a, our results showed that the scaling factor was significantly larger than one during the different stages of reading (DYSCO‐sample), which was not the case for the test–retest data (NKI‐sample). This means that the head grows significantly during both the early and advance stages of reading, and has a greater effect than the variability between scans (assessed with the test–retest scans). Hence, head growth is not negligible and important to consider in studies examining samples over these periods.

Second, we demonstrated that the PBVC computed based on volumes estimated by combining information of different time points as described in Section 2.2.2 (here, referred as longitudinal PBVC_+scaling_) leads to smaller test–retest error rates compared to the PBVC based on volumes computed at different time points independently (here, referred as cross‐sectional PBVC_+scaling_). The test–retest error assesses the ability of measuring no change on test and retest scans (i.e., two scans acquired in a short period of time in which no structural changes are expected, typically during the same scanning session). Based on the paired *T*‐test, we showed that the error rates in absolute value is significantly smaller for the longitudinal PBVC_+scaling_ compared with cross‐sectional PBVC_+scaling_ for the total cortical gray matter (see Figure 3b) and at the sub‐regions level (see Supporting Information I).

### 2.3 Statistical analysis

#### 2.3.1 Assessing the cortical development of children with typical reading skills

We measured the developmental changes in children with typical reading skills over two specific time periods: the early stage of reading (from end of kindergarten to end of second grade) and advanced stage (from end of second grade to middle of fifth grade). We performed statistical analysis on PBVC of the total gray matter volume and of the 62 cortical regions. First, we tested with a one‐sample *T*‐test if the mean PBVC_+scaling_ was different from zero in 23 TR children from the DYSCO dataset during the early stage of reading and across 20 TR children during the advanced stage of reading. The evidence for an important growth was assessed with the Bayes Factor (BF), as calculated with the “BayesFactor” package in R. The evidence was considered “substantial” for BF between 3 and 10, “strong” for BF between 10 and 100, and “decisive” for BF above 100 (Kass & Raftery, 1995).

#### 2.3.2 Assessing cortical development related to developmental dyslexia

The effect of reading groups (i.e., DR vs. TR) on the cortical development of total gray matter and the 62 regions was analyzed with linear regression modeling. We tested the full model including the reading group as a factor and gender, handedness, and family risk as confounding factors over the null model including only the confounding factors. The best fitting model was selected based on the BF, which estimates the evidence of the full model compared to the null model that included the confounding factors (Kass & Raftery, 1995). The BF was calculated with the “BayesFactor” package in R. When BF was above 3 (substantial evidence) in a certain region‐of‐interest (ROI), we performed a linear regression with the full model for that region (PBVC ~ reading group + gender + family risk + handedness) in order to quantify the effect the reading group on the PBVC in this ROI with the “lm” function from “lme4” library in R.

## 3 RESULTS

### 3.1 Brain development in TRs during primary school

**FIGURE 4:**
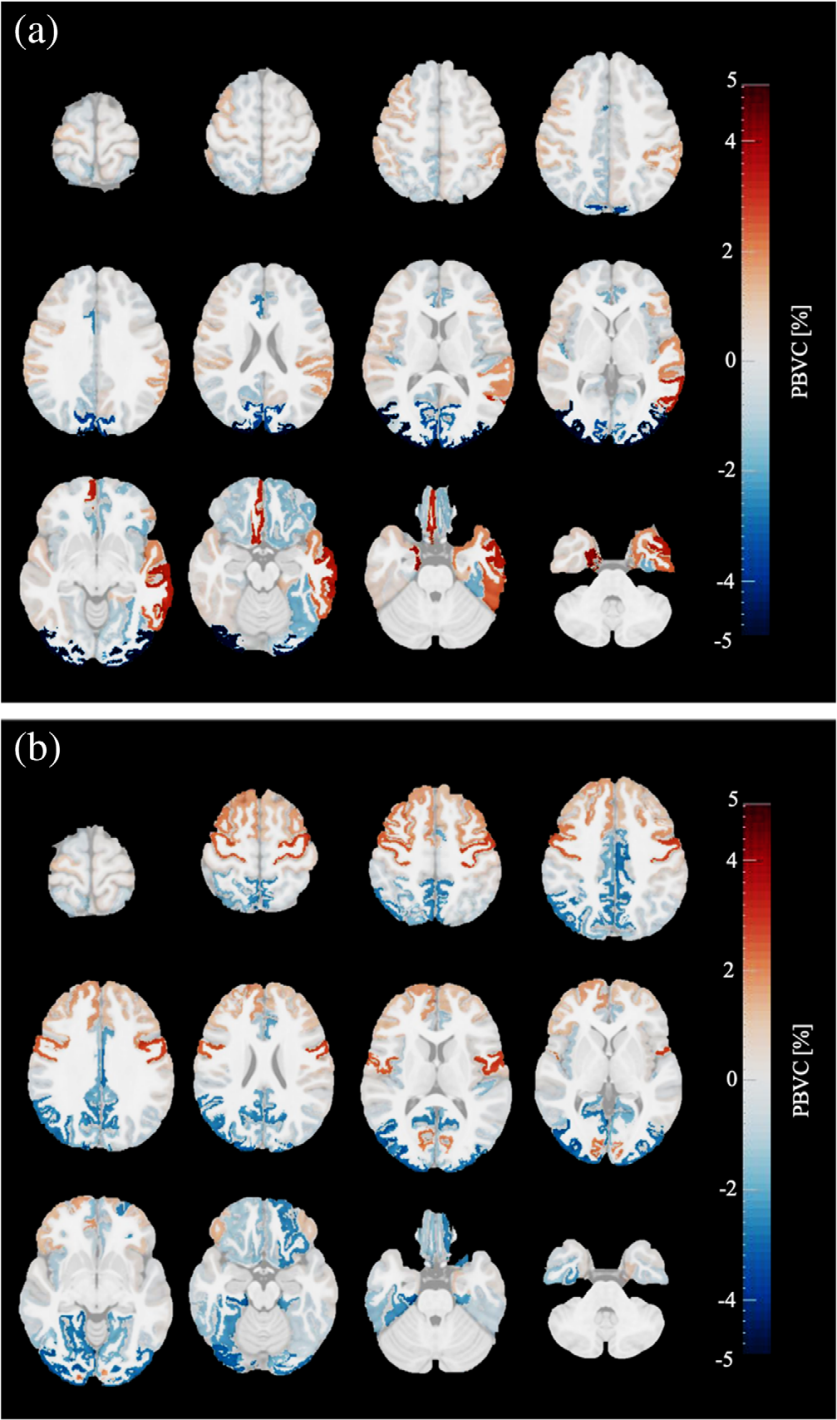
Mean percentage brain volume changes (PBVC_+scaling_) per cortical gray matter regions of children with typical reading skills occurring during a) early reading stage (*n* = 23) and (b) advanced reading stage (*n* = 20) of primary school. Red colored regions depict a volume increase and blue colored regions a volume decrease. The brighter the color, the larger the volume change; the more transparent, the lower the evidence for volume change. Images are shown in radiological convention: the left hemisphere is displayed on the right side of the image

Figure 4 depicts the PBVC_+scaling_ for each cortical gray matter sub‐region during the early reading stage (from end of kindergarten to end of second grade; Panel a) and during the advanced reading stage (from end of second grade to middle of fifth grade; Panel b). In Supporting Information II, more details are provided on the mean PBVC_+scaling_, standard deviation, and Bayesian statistics for total cortical gray matter and for each cortical sub‐region for each reading stage.

During the early reading stage, no change was observed in the total cortical gray matter. Contrary, in the cortical sub‐regions, the results demonstrated that there were substantial changes in gray matter volume. More specifically, a negative brain volume change was found with strong evidence (BF >10) in the right caudal anterior cingulate, left lateral orbitofrontal, right transverse temporal, left lingual, right pericalcarine, right precuneus and left fusiform regions, and with decisive evidence (BF >100) in bilateral cuneus and bilateral lateral occipital regions. A positive brain volume change was found with strong evidence (BF >10) in the left supramarginal region, and with decisive evidence (BF >100) in the right medial orbitofrontal, bilateral entorhinal, left inferior temporal, left middle temporal, and left superior temporal regions.

During the advanced reading stage, no change was observed in the total cortical gray matter, but again cortical sub‐regions showed changes in gray matter volume. More specifically, a negative brain volume change was found with strong evidence (BF >10) in the right isthmus cingulate, left lateral orbitofrontal, bilateral lateral occipital, left lingual, right fusiform, and right parahippocampal regions, and with decisive evidence (BF >100) in bilateral posterior cingulate, bilateral precuneus and right inferior parietal regions. A positive brain volume change was found with substantial evidence (BF >3) in right superior frontal, bilateral caudal middle frontal region, and with strong evidence (BF >10) in right precentral, and with decisive evidence (BF >100) in the left precentral regions.

### 3.2 Cortical development related to dyslexia during primary school

**FIGURE 5:**
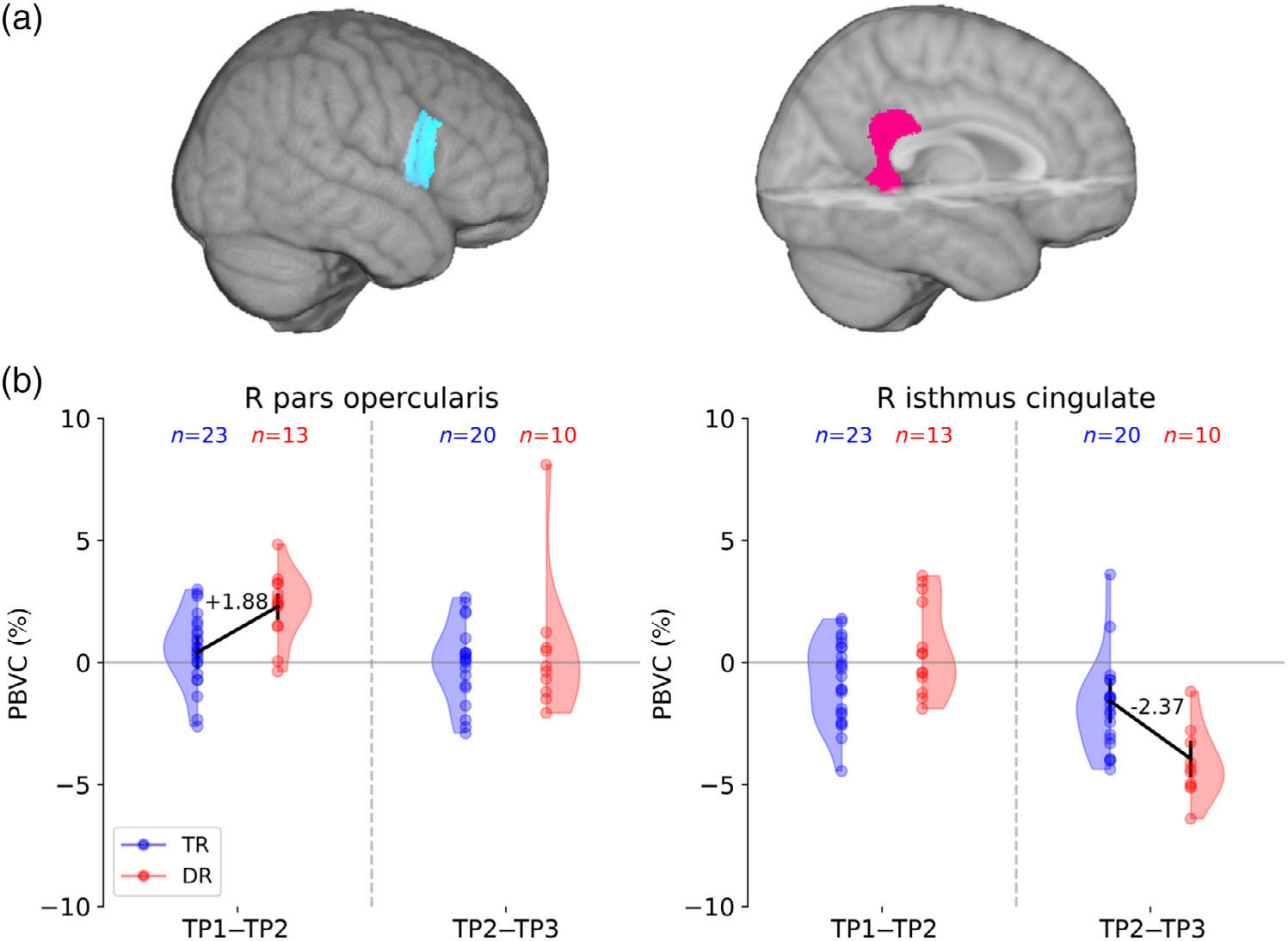
Children with dyslexia (DR) present different cortical development in the right inferior frontal and cingulate gyri compared with typical readers (TR). (a). Different cortical development patterns between DR and TR are located in the right pars opercularis and the isthmus cingulate gyri. (b). Linear regression models showing a positive effect of dyslexia on the percentage brain volume change (PBVC_+scaling_) in the right pars opercularis gyrus during early primary school (TP1–TP2) and a negative effect of dyslexia in the right isthmus cingulate gyrus during late primary school (TP2–TP3)

At the whole‐brain level, no evidence for an effect of reading group on the PBVC_+scaling_ over the confounding factors (i.e., age, gender, handedness, and family risk) was found in the cortical gray matter. At the sub‐regions level, strong evidence for the effect of the reading group was found in the right pars opercularis (BF = 26.79) during the early stage of reading, with a larger increase in gray matter volume for DR than for TR (see Figure 5 **)**. During the advanced stage of reading, strong evidence for a group difference in cortical development was found in the right isthmus cingulate (BF = 16.21) with a larger decrease in gray matter volume in DR than in TR. The effects as estimated with the linear regression model are shown in Table 2. In other cortical regions, no substantial evidence of reading group has been found over confounding factors (see Supporting Information II).

**TABLE 2:** Effect of developmental dyslexia on the cortical development assessed with (Bayesian) linear regression

ROI	Intercept	Effect of dyslexia	*t*‐value	*p*‐value	BF
	Estimate ± *SE*	Estimate ± *SE*			
*Early primary school*					
R. pars opercularis	0.41 ± 0.68	+1.88 ± 0.55	3.399	.002	26.79
*Late primary school*					
R. isthmus cingulate	−1,57 ± 0.91	−2.37 ± 0,74	−3.205	.004	16.21

*Note*: The Bayes Factor (BF) represents the probability in favor of the full model (including reading group as a factor in addition to the confounding factors) over the null model (including only the confounding factors: gender, handedness, and family risk).

## 4 DISCUSSION

In the current study, we acquired T1 MRI data at three time points to investigate neurodevelopment during early and advanced reading stages in children with typical reading skills and children with dyslexia. We reliably measured the cortical change over time by using the longitudinal PBVC considering head growth, PBVC_+scaling_. Our results showed that in typical brain development different regions co‐develop during specific periods of primary school. The early reading stage is mainly characterized by volumes increases in left temporo(parietal) regions and decreases in occipital cortex, and the advanced reading stage is by volume increases in frontal regions and decreases in occipital and parietal regions. In addition, atypical neurodevelopmental trajectories related to dyslexia were found outside the reading network, namely in right IFG opercularis and right isthmus cingulate.

### 4.1 Typical brain development during early and advanced reading stages

By measuring structural changes during specific reading stages, we could determine how the maturation of the cortical gray matter occurs over primary school. During the early and advanced stages of reading, our results showed that total cortical gray matter (at the tissue level) is stable. These results are in line with previous longitudinal studies on cortical development, showing a relative stable gray matter volume during primary school, and only from adolescence onward a volume decrease (Lebel & Beaulieu, 2011; Mills et al., 2016; Sowell et al., 2003). In combination with infants studies showing clear gray matter volume increases during the first years of life (Deoni, Dean, Remer, Dirks, & O'Muircheartaigh, 2015; Natu et al., 2019), this indicates that cortical maturation from infancy to adulthood is at the macroscopic level best characterized by an inverted U‐shape development. Although no direct relationship can be established between changes in gray matter volume and microscopic changes in neural cells, the timing of cortical maturation observed at the macroscopic level were suggested to coincide with the synaptic development observed at the microscopic level in histological studies (Huttenlocher & Dabholkar, 1997; Petanjek et al., 2011). At the microscopic level, cortical maturation is associated with the generation of excess synaptic connections, their selective elimination and later on, the strengthening of functional connections (Neniskyte & Gross, 2017). This maturation would at the macroscopic level be reflected by an inverted U‐shape with first a volume increase (corresponding to a positive PBVC_+scaling_), then reaching peak volume (PBVC_+scaling_ close to 0), followed by switching to a phase of volume decrease (corresponding to a negative PBVC_+scaling_).

While the cortical gray matter does not change at the whole‐brain level during primary school, local changes occur in specific cortical sub‐regions. Assuming that cortical maturation is following an inverted U‐shape at the macroscopic level, regions that display volume increase would be in an earlier phase of maturation while regions with volume decrease would be in a later stage of maturation. We observed that in the first 2 years of primary school the occipital regions forming the primary visual (i.e., pericalcarine) and associative visual (i.e., lateral occipital, cuneus, and lingual) cortex, display volume decrease (negative PBVC_+scaling_), suggesting that the volume peak has already been reached before primary school and that they are in a later stage of maturation. This is also in line with previous studies based on neuroimaging and histology, showing cortical thinning or volume decrease in the visual cortex during childhood (Aubert‐Broche et al., 2013; Brown & Jernigan, 2012; Gogtay & Thompson, 2010; Natu et al., 2019). In the late years of primary school (end of grade 2–middle of grade 5), we observed further volume decreases in the associative visual cortex (i.e., lingual and lateral occipital). The primary visual (i.e., pericalcarine) and primary auditory cortex (i.e., transverse temporal) showed only a volume decrease during the early but not during the advance stage of reading, suggesting a finished maturation of perceptual regions by the end of grade 2.

In contrast to volume decreases, we observed during the first 2 years of reading development gray matter volume increase in left supramarginal gyrus, left inferior, middle and superior temporal gyrus, which are regions belonging to what is typically defined as the reading network. In addition, during that same period, also volume increases were observed in bilateral entorhinal cortex, a region which was recently be suggested to be specifically important for reading skills, namely for learning to couple a referent to its written word form (Liuzzi, Brufaerts, & Vandenberghe, 2019). This means that key regions of the reading network co‐develop and are in early stage of maturation (presented here as an increase of brain volume) at a period in which children learn to read and write, while the volume of these regions stabilizes during the advanced stage of reading when explicit reading instruction moves more to the background. This could indicate that there is a strengthening of the reading network in close interaction with the environmental input. This is in line with the interactive specialization theory (Johnson, 2011), hypothesizing that a new cognitive skills, such as reading, is not just the unidirectional result of brain maturation, but is rather the result of a complex and dynamic interaction between brain and environment. Given that we observed that plasticity of the left reading network occurs especially during the early stage of reading (i.e., volume increase), with a plateau reached after grade 2, this might argue for early reading intervention in children with (a risk for) reading difficulties. In current clinical practice dyslexia is typically only diagnosed after years of reading failure, resulting in reading intervention that is only started after grade 2 (Ozernov‐Palchik & Gaab, 2016). Our results on plasticity in the left reading network during the first years of reading provide a biological explanation of why preventive and early reading interventions are more effective than interventions provided during the later grades of primary school (Wanzek & Vaughn, 2007). During the advanced reading stage, volume increases are only observed in frontal (i.e., precentral, superior frontal, and caudal middle frontal) regions. Given that frontal processing is associated to higher order cognitive and attention executive functions (Shaw et al., 2006), maturation in these regions could support solving more complex arithmetic problems (Butterworth & Walsh, 2011) and advanced reading (Krishnan, Watkins, & Bishop, 2016).

### 4.2 Different developmental pace in dyslexia is located outside the reading network

Studies on developmental dyslexia frequently reported structural differences in the left inferior frontal, temporo‐parietal, and occipito‐temporal regions (Linkersdörfer et al., 2012; Richlan, Kronbichler, & Wimmer, 2011) with some studies showing that those differences already appeared at a prereading age (Beelen et al., 2019; Clark et al., 2014; Im, Raschle, Smith, Ellen Grant, & Gaab, 2016; Raschle, Chang, & Gaab, 2011). However, these findings were not consistently found across all studies (Ramus, Altarelli, Jednoróg, Zhao, & di Covella, 2017). One explanation could be that those differences are not persistent over time, involving dynamic mechanisms leading to variable group differences depending on the observed time frame (Yeatman, Dougherty, Ben‐Shachar, & Wandell, 2012). Given that studies investigating gray matter trajectories in dyslexia are lacking, we examined cortical development during both the early and advanced stages of reading in children with dyslexia and typical reading skills. Our results showed that different neural trajectories were found in the right pars opercularis and the right isthmus cingulate gyri, but within the left hemispheric reading network, structural development was not different between reading groups. In a previous study, we have shown on the same cohort that children with dyslexia have smaller fusiform gyri than TRs before starting primary school (Beelen et al., 2019). Findings of the current study indicate that these structural differences related to dyslexia are persistent across primary school since the fusiform did not display deviant development during the early and advanced stage of reading.

Concerning the higher volume increase in the right pars opercularis during early stage of reading in children with dyslexia, previous studies based on functional neuroimaging demonstrated that regions in the right hemisphere, such as the inferior frontal gyrus, are hyper‐activated in DRs (Eden et al., 2004; Shaywitz, Lyon, & Shaywitz, 2006). This hyper‐activation was interpreted as a compensatory mechanism to an impaired recruitment of its left counterpart, which is involved in phonological processing, speech planning, lexical access, semantics, attention and inhibition (Pugh et al., 2001; Yu et al., 2018). It is possible that the increased recruitment of the right inferior frontal gyrus could have led to a higher volume increase in children with dyslexia compared to children with typical reading skills, although this can only be validated from correlation between brain functions and structure. Longitudinal studies on white matter also corroborate a compensatory function for the right pars opercularis as they found that children at risk for dyslexia who subsequently become good readers have a faster white matter development in the right Superior Longitudinal Fasciculus, a tract which makes a connection from inferior parietal to the pars opercularis (Wang et al., 2016). However, while in that study right white matter increase seems to be an efficient neural compensatory mechanism to overcome reading problems, our findings suggest that gray matter increase in right pars opercularis does not lead to typical reading skills, since our dyslexic sample has persisting poor reading abilities throughout primary school.

During the advanced stage of reading development, we observed a larger volume reduction in the right isthmus cingulate in children with dyslexia. Although this region is typically not considered a core reading region, activation in the posterior cingulate has been associated with the imageability of words during reading tasks, hence with its semantic aspects (Graves et al., 2014). It is possible that readers with dyslexia who have deficit in phonological processing, learn to read by using systems relying more on semantic processing (Shaywitz et al., 2007), although this is a less efficient strategy for reading (Siegelman et al., 2020; Woollams, Lambon Ralph, Madrid, & Patterson, 2016).

### 4.3 Contributions, limitations, and future perspectives

In the current study, we analyzed longitudinal data to assess brain development throughout primary school. Longitudinal designs are more appropriate to differentiate the interindividual from the intraindividual variability and require fewer participants compared to cross‐sectional designs in order to detect small differences in brain structures (Steen, Hamer, & Lieberman, 2007). Here, the proposed measure, PBVC_+scaling_, measures the structural change from longitudinal data at the image level, which enables to capture individual differences without assuming any model. Indeed, the linear and nonlinear changes are measured from the affine registration of the head images and the non‐rigid registration of the brain images at two different time points. The PBVC was adapted to pediatric data by modulating it with a scaling factor of the head growth (PBVC_+scaling)_. In Section 2, we demonstrated that the proposed measure was more appropriate to assess brain development in children. Indeed, the head grows significantly during early and advanced stage of reading development and that the measurement of PBVC is more reliable with the longitudinal method than with the traditional cross‐sectional method.

In previous longitudinal studies, the common approach for analyzing longitudinal data of children was to conduct the MRI analyses at each time independently and to combine these data points at the statistical level using a mixed effect model (Phan et al., 2017). The issue is that the statistical approach requires an assumption regarding the model underlying the development (e.g., linear, quadratic, and cubic), which is dependent on the variables included in the model, especially the age range of the sample (Fjell et al., 2010). This can also lead to comparisons of groups with developmental trajectories differently modeled, which makes statistical testing not straightforward (Mills & Tamnes, 2014). Contrary to the statistical estimation of developmental trajectory curves, the PBVC_+scaling_ directly measures neurodevelopmental changes. By consequence, the developmental change can be directly compared between groups for studying differences in brain development. The advantage is that the PBVC_+scaling_ is fully automated and directly calculates the developmental change from the image with smaller error rates compared to the traditional cross‐sectional method. This enables big data analysis and potentially analyses at the individual level. The current error rate might be still too high to perceive subtle structural changes in shorter time period or in small sub‐regions but could still be improved with a better parameter setting or more advanced segmentation methods, as well as analyzing images with higher resolution.

Finally, although our study provides the first insights in regional gray matter development in typical and DRs across primary school, it is evident that our findings need replication in large‐scale longitudinal studies. The observed effects in the 62 regions were not corrected for multiple comparison since it is debated whether Bayesian statistics require multiple comparison correction and how this should be implemented (Dienes, 2014; Keysers, Gazzola, & Wagenmakers, 2020). Yet, the advantage of a BF (in contrast to *p*‐values) is that it allows a straightforward interpretation with larger deviations from 1 providing stronger evidence. Our findings on neurodevelopment in the reading network and on dyslexia‐related differences are based a BF above 10, which is often recommended for more exploratory research (Keysers et al., 2020), hence we provided strong evidence for our conclusions. Nevertheless, future large‐scale studies remain vital for generalization and for investigating how individual differences in reading‐related behavioral tasks relate to cortical development. Due to our limited sample size, we could only make conclusions at the group level (TRs vs. DRs) rather than characterizing brain development in relation to the specific reading tasks. Yet, we acknowledge that mapping the brain with the behavioral component of reading would be interesting, particularly to understand neural compensation in children with dyslexia.

## 5 CONCLUSIONS

The aim of this study was to assess the typical and atypical cortical development during specific stages of reading. By applying a longitudinal measure that captures the structural volume change directly at the image level while considering the head growth (PBVC_+scaling_), we used a child‐adjusted and reliable measure to track cortical changes. The examination of typical cortical development showed different groups of co‐developing regions during the early versus advanced reading stages, with volume increases in the left reading network taking place during the early stage of reading, after which it reaches a plateau. This suggests more plasticity in the reading network during the first years of primary school, hence this might urge for preventive and early interventions. With regard to dyslexia, we observed atypical neural trajectories in regions not belonging to what is typically considered the reading network (i.e., right pars opercularis and isthmus cingulate regions), hence this might represent compensatory mechanisms.

## CONFLICT OF INTEREST

There is no conflict of interest to declare.

## Supporting information

**Appendix** S1: Supporting Information

## Data Availability

This study was approved by the Ethical Committee of the University Hospital of Leuven [B322201214607] and for all participants, informed consent was obtained from their parents according to the Declaration of Helsinki. The conditions of our ethics approval do not permit public archiving of anonymised study data, since consent had only been obtained for the participation in the study, and not to share data with third parties. Researchers seeking access to the study data should contact the last author (maaike.vandermosten@kuleuven.be) explaining the purpose of their request. In accordance with the EU general data protection regulation (GDPR), data will be released to requestors upon the following conditions: consent of the representative of the minor and a formal agreement between parties. Please note that the MRI data cannot be shared under any circumstance, as MRI data are person‐specific and therefore cannot be considered anonymous.
