# Supplementary material for: Structural brain dynamics across reading development: A longitudinal MRI study from kindergarten to grade 5
Source: Hum Brain Mapp. 2021 Jul 1;42(14):4497–509. doi: 10.1002/hbm.25560 (PMC8410537; doi:10.1002/hbm.25560)
Supplement: Supplementary file 1 — Appendix S1: Supporting Information [file HBM-42-4497-s001.zip › HBM_25560_HBM_25560_Supplementary1_test_retest_format.pdf]

CGM region		T-test: H0: PBVC long abs. error >= PBVC cross abs. error / H1: PBVC long abs. erro < PBVC cross abs					
		**PBVC long Abs. error Mean ± STD**	**PBVC cross Abs. error Mean ± STD**	**df**	**T-value**	**P-value**	**Corrected p-value**
CACg L	L. Caudal Anterior Cingulate	0.90 ± 0.97	17.95 ± 19.29	69	-7,429	1.11 e-10	2.23 e-10
CACg R	R. Caudal Anterior Cingulate	0.66 ± 0.62	20.01 ± 17.40	69	-9,310	4.00 e-14	8.56 e-14
CMF L	L. Caudal Middle Frontal	1.11 ± 1.70	11.65 ± 0.78	69	-9,350	3.46 e-14	7.66 e-14
CMF R	R. Caudal Middle Frontal	0.97 ± 0.88	8.45 ± 7.78	69	-9,010	1.41 e-13	2.49 e-13
ICg L	L. Isthmus Cingulate	1.04 ± 1.07	11.84 ± 9.09	69	-9,880	3.87 e-15	1.50 e-14
ICg R	R. Isthmus Cingulate	1.27 ± 1.27	11.27 ± 8.28	69	-10,180	1.10 e-15	5.92 e-15
PCg L	L. Posterior Cingulate	0.95 ± 1.22	10.44 ± 7.97	69	-9,813	5.04 e-15	1.84 e-14
PCg R	R. Posterior Cingulate	1.02 ± 1.50	12.68 ± 9.79	69	-10,123	1.41 e-15	6.71 e-15
RACg L	L. Rostral Anterior Cingulate	0.99 ± 1.02	18.52 ± 14.77	69	-9,960	2.78 e-15	1.15 e-14
RACg R	R. Rostral Anterior Cingulate	1.10 ± 0.94	22.80 ± 18.86	69	-9,590	1.25 e-14	4.29 e-14
LOF L	L. Lateral Orbitofrontal	1.36 ± 1.56	5.47 ± 5.30	69	-7,200	2.89 e-10	2.93 e-10
LOF R	R. Lateral Orbitofrontal	1.30 ± 1.53	5.08 ± 3.98	69	-7,655	4.27 e-11	4.56 e-11
MOF L	L. Medial Orbitofrontal	1.71 ± 1.56	11.33 ± 10.32	69	-7,744	2.93 e-11	3.25 e-11
MOF R	R. Medial Orbitofrontal	1.64 ± 1.53	11.59 ± 8.93	69	-9,171	7.29 e-14	1.48 e-13
PrCt L	L. Paracentral	1.07 ± 0.94	9.27 ± 8.91	69	-7,704	3.49 e-11	3.79 e-11
PrCt R	R. Paracentral	1.20 ± 0.79	8.66 ± 7.25	69	-8,860	2.68 e-13	4.26 e-13
POper L	L. Pars Opercularis	0.76 ± 0.77	12.04 ± 10.33	69	-9,471	2.09 e-14	5.20 e-14
POper R	R. Pars Opercularis	0.80 ± 0.85	8.77 ± 7.02	69	-9,433	2.45 e-14	5.84 e-14
POrb L	L. Pars Orbitalis	1.76 ± 1.50	12.38 ± 10.25	69	-8,851	2.79 e-13	4.32 e-13
POrb R	R. Pars Orbitalis	1.72 ± 1.84	11.95 ± 8.23	69	-10,535	2.61 e-16	5.06 e-15
PTri L	L. Pars Triangularis	1.28 ± 1.36	11.71 ± 10.76	69	-8,113	6.22 e-12	7.72 e-12
PTri R	R. Pars Triangularis	1.13 ± 1.80	11.26 ± 9.68	69	-9,518	1.72 e-14	4.96 e-14
RMF L	L. Rostral Middle Frontal	1.29 ± 1.87	8.20 ± 6.41	69	-9,005	1.46 e-13	2.52 e-13
RMF R	R. Rostral Middle Frontal	1.22 ± 1.73	7.48 ± 6.45	69	-7,847	1.91 e-11	2.15 e-11
SF L	L. Superior Frontal	1.12 ± 1.36	6.43 ± 5.59	69	-7,906	1.49 e-11	1.71 e-11
SF R	R. Superior Frontal	1.00 ± 1.12	6.60 ± 5.19	69	-9,501	1.84 e-14	4.96 e-14
Cn L	L. Cuneus	0.95 ± 0.77	10.71 ± 7.83	69	-1,032	6.19 e-16	5.48 e-15

CGM region		T-test: H0: PBVC long abs. error >= PBVC cross abs. error / H1: PBVC long abs. erro < PBVC cross abs					
		**PBVC long Abs. error Mean ± STD**	**PBVC cross Abs. error Mean ± STD**	**df**	**T-value**	**P-value**	**Corrected p-value**
Cn R	R. Cuneus	0.91 ± 0.79	15.45 ± 11.64	69	-10,380	4.89 e-16	5.06 e-15
LO L	L. Lateral Occipital	1.51 ± 1.62	8.48 ± 5.99	69	-9,470	2.10 e-14	5.20 e-14
LO R	R. Lateral Occipital	1.31 ± 1.27	7.88 ± 5.98	69	-8,975	1.65 e-13	2.77 e-13
Ling L	L. Lingual	1.22 ± 1.13	7.55 ± 6.21	69	-8,573	8.96 e-13	1.23 e-12
Ling R	R. Lingual	0.77 ± 0.72	8.68 ± 5.64	69	-1,178	2.2 e-16	1.09 e-16
PCal L	L. Pericalcarine	1.66 ± 1.87	13.48 ± 11.46	69	-8,262	3.32 e-12	4.38 e-12
PCal R	R. Pericalcarine	1.28 ± 1.22	11.87 ± 8.71	69	-10,437	3.88 e-16	5.06 e-15
IP L	L. Inferior Parietal	0.97 ± 0.79	7.03 ± 5.14	69	-10,099	1.55 e-15	6.87 e-15
IP R	R. Inferior Parietal	1.05 ± 1.12	5.96 ± 5.65	69	-7,060	5.19 e-10	5.19 e-10
PstCt L	L. Postcentral	0.88 ± 1.01	7.09 ± 5.36	69	-9,558	1.45 e-14	4.50 e-14
PstCt R	R. Postcentral	0.88 ± 0.89	6.35 ± 5.36	69	-8,610	7.68 e-13	1.08 e-12
PrCt L	L. Precentral	1.09 ± 1.09	5.55 ± 4.16	69	-8,709	5.06 e-13	7.30 e-13
PrCt R	R. Precentral	0.97 ± 0.80	4.94 ± 3.81	69	-8,837	2.95 e-13	4.47 e-13
PCn L	L. Precuneus	0.91 ± 0.91	8.31 ± 6.09	69	-10,226	9.21 e-16	5.92 e-15
PCn R	R. Precuneus	0.90 ± 0.86	9.01 ± 6.82	69	-10,217	9.55 e-16	5.92 e-15
SP L	L. Superior Parietal	1.16 ± 1.06	7.29 ± 6.35	69	-8,160	5.18 e-12	6.69 e-12
SP R	R. Superior Parietal	1.20 ± 1.21	6.74 ± 4.45	69	-10,178	1.12 e-15	5.92 e-5
SMg L	L. Supramarginal	0.97 ± 1.08	7.16 ± 4.77	69	-10,687	2.2 e-16	4.36 e-15
SMg R	R. Supramarginal	0.89 ± 0.89	6.84 ± 4.94	69	-10,173	1.15 e-15	5.92 e-15
Ent L	L. Entorhinal	1.42 ± 1.38	16.36 ± 15.65	69	-7,949	1.24 e-11	1.45 e-11
Ent R	R. Entorhinal	1.67 ± 1.54	13.77 ± 10.84	69	-9,402	2.78 e-14	6.39 e-14
Fusif L	L. Fusiform	1.18 ± 1.24	7.21 ± 5.84	69	-8,718	4.87 e-13	7.19 e-13
Fusif R	R. Fusiform	1.18 ± 1.23	6.92 ± 5.35	69	-9,167	7.41 e-14	1.48 e-13
IT L	L. Inferior Temporal	1.90 ± 2.16	8.31 ± 5.89	69	-8,966	1.72 e-13	2.81 e-13
IT R	R. Inferior Temporal	1.50 ± 1.57	7.19 ± 6.26	69	-8,019	9.23 e-12	1.12 e-11
MT L	L. Middle Temporal	1.04 ± 1.18	7.31 ± 4.95	69	-1,039	4.79 e-16	5.06 e-15
MT R	R. Middle Temporal	1.01 ± 0.85	5.95 ± 5.31	69	-7,547	6.73 e-11	7.07 e-11

CGM region		T-test: H0: PBVC long abs. error >= PBVC cross abs. error / H1: PBVC long abs. erro < PBVC cross abs					
		**PBVC long Abs. error Mean ± STD**	**PBVC cross Abs. error Mean ± STD**	**df**	**T-value**	**P-value**	**Corrected p-value**
PrHp L	L. Parahippocampal	1.53 ± 1.37	10.48 ± 8.27	69	-9,067	1.13 e-13	2.12 e-13
PrHp R	R. Parahippocampal	1.62 ± 1.63	10.77 ± 7.78	69	-9,504	1.82 e-14	4.96 e-14
ST L	L. Superior Temporal	0.85 ± 1.02	5.51 ± 5.03	69	-8,014	9.42 e-12	1.12 e-11
ST R	R. Superior Temporal	1.08 ± 1.34	5.25 ± 4.19	69	-8,534	1.06 e-12	1.43 e-12
TT L	L. Transverse Temporal	1.21 ± 1.34	13.38 ± 12.74	69	-8,142	5.49 e-12	6.94 e-12
TT R	R. Transverse Temporal	1.40 ± 1.35	14.02 ± 11.55	69	-9,019	1.38 e-13	2.49 e-13
Ins L	L. Insula	0.77 ± 0.64	7.13 ± 5.65	69	-9,582	1.32 e-14	4.29 e-14
Ins R	R. Insula	0.82 ± 0.75	5.90 ± 4.55	69	-9,142	8,.25 e-14	1.60 e-13
