# Supplementary material for: Structural brain dynamics across reading development: A longitudinal MRI study from kindergarten to grade 5
Source: Hum Brain Mapp. 2021 Jul 1;42(14):4497–509. doi: 10.1002/hbm.25560 (PMC8410537; doi:10.1002/hbm.25560)
Supplement: Supplementary file 1 — Appendix S1: Supporting Information [file HBM-42-4497-s001.zip › HBM_25560_HBM_25560_Supplementary2_brain_development_analysis.pdf]

	TR: Cortical development (TP1-TP2)		TR: Cortical development (TP2-TP3)		
**ROI**	**PBVC [%] - TR** n=23 **mean (std)**	**T-test: PBVC !=0** **BF**	**PBVC [%] - TR** n=20 **mean (std)**	**T-test: PBVC !=0** **BF**	
Whole Brain	+1,88 (0,72)	546100937,00	+1,00 (0,71)	4019,96	
Cortical Gray Matter	+0,18 (1,22)	0,27	-0,11 (1,56)	0,24	
Deep Gray Matter	-0,24 (1,26)	0,32	-0,48 (1,63)	0,50	
White Matter	+5,10 (2,45)	8981751,00	+2,97 (2,78)	213,59	
L. Caudal Anterior Cingulate	-0,59 (2,66)	0,36	-1,93 (3,43)	2,77	
R. Caudal Anterior Cingulate	-2,40 (3,37)	16,12	+0,90 (2,58)	0,65	
L. Caudal Middle Frontal	+0,15 (2,24)	0,23	+1,47 (2,55)	3,12	
R. Caudal Middle Frontal	+1.03 (2.79)	0,85	+1,80 (3,02)	3,65	
L. Rostral Anterior Cingulate	-1.43 (3,34)	1,28	-1,12 (2,23)	1,74	
R. Rostral Anterior Cingulate	-1,52 (2,98)	2,50	-0,55 (1,75)	0,55	
L. Lateral Orbitofrontal	-1,39 (2.05)	11,76	-2,40 (2,64)	53,25	
R. Lateral Orbitofrontal	-0,96 (2,13)	1,57	-1,01 (2,01)	1,76	
L. Medial Orbitofrontal	-1,44 (2,37)	5,88	-1,00 (2,81)	0,68	
R. Medial Orbitofrontal	+2,94 (1,74)	293289,90	-1,39 (2,61)	2,19	
L. Paracentral	+0,30 (2,85)	0,25	-0,25 (3,18)	0,25	
R. Paracentral	-0,40 (1,94)	0,34	-0,05 (2,98)	0,23	
L. Pars Opercularis	-0,49 (2,45)	0,33	+0,09 (2,32)	0,24	
R. Pars Opercularis	+0,53 (1,48)	0,76	-0,02 (1,61)	0,23	
L. Pars Orbitalis	-1,67 (3,27)	2,46	+0,75 (3,41)	0,36	
R. Pars Orbitalis	-0,25 (3,30)	0,23	+1,10 (3,76)	0,49	
L. Pars Triangularis	+1,12 (4,24)	0,45	-0,04 (3,26)	0,23	
R. Pars Triangularis	+0,40 (3,12)	0,26	+0,93 (2,62)	0,68	
L. Rostral Middle Frontal	-0,32 (2,38)	0,26	+0,83 (2,71)	0,53	
R. Rostral Middle Frontal	+0,40 (1,79)	0,37	+1,00 (2,17)	1,30	
L. Superior Frontal	+0,03 (1,34)	0,22	+0,89 (1,71)	2,02	

ROI	TR: Cortical development (TP1-TP2)		TR: Cortical development (TP2-TP3)		
	**PBVC [%] - TR** n=23 **mean (std)**	**T-test: PBVC !=0** **BF**	**PBVC [%] - TR** n=20 **mean (std)**	**T-test: PBVC !=0** **BF**	
R. Superior Frontal	-0,31 (1,75)	0,31	+1,39 (2,17)	5,30	
L. Precentral	+0,12 (2,78)	0,22	+2,52 (2,53)	111,34	
R. Precentral	+0,80 (1,99)	1,07	+2,18 (2,56)	30,98	
L. Cuneus	-3,55 (3,17)	1069,51	-1,26 (3,20)	0,85	
R. Cuneus	-3,56 (1,93)	1253309,00	-1,32 (2,37)	2,68	
L. Lateral Occipital	-5,18 (3,00)	430584,10	-2,76 (2,87)	83,83	
R. Lateral Occipital	-6,57 (3,08)	13401364,00	-3,06 (3,74)	23,40	
L. Lingual	-0,91 (1,37)	10,19	-1,71 (2,17)	17,95	
R. Lingual	-0,25 (1,18)	0,35	-2,61 (2,07)	1201,00	
L. Pericalcarine	-2,73 (4,86)	3,89	+1,76 (4,66)	0,78	
R. Pericalcarine	-3,30 (4,66)	15,48	+1,95 (5,30)	0,73	
L. Inferior Parietal	-0,03 (1,85)	0,22	-0,72 (2,68)	0,44	
R. Inferior Parietal	-0,54 (2,14)	0,42	-2,57 (2,28)	374,56	
L. Postcentral	+0,33 (2,02)	0,29	+0,67 (2,21)	0,51	
R. Postcentral	+0,14 (1,63)	0,24	+0,31 (2,19)	0,28	
L. Precuneus	+0,35 (1,81)	0,32	-2,49 (2,26)	286,02	
R. Precuneus	-1,11 (1,53)	18,65	-2,43 (2,29)	208,16	
L. Superior Parietal	-0,63 (1,99)	0,59	-0,36 (3,05)	0,26	
R. Superior Parietal	-0,84 (1,48)	3,96	-1,63 (2,86)	2,98	
R. Supramarginal	+0,86 (2,41)	0,77	+0,42 (2,20)	0,32	
L. Supramarginal	+1,31 (1,69)	30,29	+0,17 (1,95)	0,25	
L. Isthmus Cingulate	-0,12 (3,24)	0,22	-1,50 (3,98)	0,77	
R. Isthmus Cingulate	-0,76 (1,67)	1,61	-1,83 (1,93)	72,86	
L. Posterior Cingulate	-0,04 (1,87)	0,22	-2,62 (1,65)	18766,20	
R. Posterior Cingulate	-0,53 (2,39)	0,36	-1,67 (1,63)	143,22	

	TR: Cortical development (TP1-TP2)		TR: Cortical development (TP2-TP3)		
ROI	**PBVC [%] - TR**<br>n=23<br>**mean (std)**	**T-test: PBVC !=0**<br>**BF**	**PBVC [%] - TR**<br>n=20<br>**mean (std)**	**T-test: PBVC !=0**<br>**BF**	
L. Entorhinal	+1,80 (1,90)	174,40	+,069 (1,91)	0,72	
R. Entorhinal	+3,56 (2,89)	3464,90	+0,17 (2,59)	0,24	
L. Fusiform	-1,67 (2,01)	53,11	-0,65 (2,12)	0,52	
R. Fusiform	+0,26 (1,83)	0,27	-2,20 (3,04)	10,41	
L. Inferior Temporal	+2,18 (1,73)	4618,33	-0,76 (1,64)	1,34	
R. Inferior Temporal	+0,48 (1,99)	0,40	-1,30 (2,50)	2,02	
L. Middle Temporal	+3,01 (1,58)	2075897,00	+0,10 (1,65)	0,24	
R. Middle Temporal	+0,15 (2,13)	0,23	-0,47 (1,74)	0,44	
L. Parahippocampal	+0,82 (2,48)	0,64	-2,11 (1,96)	229,02	
R. Parahippocampal	-0,57 (2,79)	0,33	-2,85 (3,83)	12,20	
L. Superior Temporal	+1,55 (1,72)	109,48	0,16 (1,90)	0,25	
R. Superior Temporal	+0,67 (2,16)	0,57	-0,08 (2,06)	0,24	
L. Transverse Temporal	-1,04 (2,85)	0,81	-1,37 (2,94)	1,37	
R. Transverse Temporal	-1,90 (2,70)	14,73	-0,68 (1,98)	0,63	
L. Insula	-0,50 (2,26)	0,36	0,18 (2,22)	0,25	
R. Insula	-0,48 (2,86)	0,29	-0,86 (2,69)	0,56	

**Legend**	
	TR = typical readers
	DR = dyslexic reader
	TP1 = end of kindergarten
	TP2 = end of grade 2
	TP3 = mid grade 5

	TP1-TP2: Difference in change between TR and DR	TP2-TP3: Difference in change between TR and DR
Full model: PBVC ~ group + gender + family risk + handedness<br>Null model: PBVC ~ gender + family risk + handedness		
**ROI**	**BF**	**BF**
Whole Brain	0,351	0,47
Cortical Gray Matter	0,956	0,50
Deep Gray Matter	0,350	0,67
White Matter	0,509	0,37
L. Caudal Anterior Cingulate	0,677	0,38
R. Caudal Anterior Cingulate	0,370	0,61
L. Caudal Middle Frontal	0,478	0,47
R. Caudal Middle Frontal	0,815	0,34
L. Rostral Anterior Cingulate	0,370	0,35
R. Rostral Anterior Cingulate	0,375	2,35
L. Lateral Orbitofrontal	0,360	0,37
R. Lateral Orbitofrontal	0,357	0,38
L. Medial Orbitofrontal	0,470	1,15
R. Medial Orbitofrontal	0,556	0,43
L. Paracentral	1,891	0,89
R. Paracentral	0,422	0,73
L. Pars Opercularis	0,471	0,37
R. Pars Opercularis	**26,788**	0,42
L. Pars Orbitalis	0,747	0,50
R. Pars Orbitalis	0,350	0,66
L. Pars Triangularis	0,381	0,41
R. Pars Triangularis	0,791	0,45
L. Rostral Middle Frontal	0,610	0,61
R. Rostral Middle Frontal	0,391	0,44
L. Superior Frontal	1,718	0,63

	TP1-TP2: Difference in change between TR and DR	TP2-TP3: Difference in change between TR and DR
Full model: PBVC ~ group + gender + family risk + handedness<br>Null model: PBVC ~ gender + family risk + handedness		
**ROI**	**BF**	**BF**
R. Superior Frontal	0,606	0,54
L. Precentral	1,068	1,67
R. Precentral	2,823	0,39
L. Cuneus	0,499	0,36
R. Cuneus	0,513	0,37
L. Lateral Occipital	0,541	0,53
R. Lateral Occipital	0,568	0,37
L. Lingual	0,507	0,39
R. Lingual	0,364	0,39
L. Pericalcarine	0,511	0,43
R. Pericalcarine	0,449	0,46
L. Inferior Parietal	0,929	0,39
R. Inferior Parietal	0,390	0,49
L. Postcentral	0,973	2,22
R. Postcentral	1,037	1,05
L. Precuneus	0,870	0,40
R. Precuneus	0,753	0,36
L. Superior Parietal	0,420	0,43
R. Superior Parietal	0,361	0,45
R. Supramarginal	1,048	0,42
L. Supramarginal	0,429	0,69
L. Isthmus Cingulate	0,675	1,06
R. Isthmus Cingulate	1,419	16,21
L. Posterior Cingulate	1,008	0,46
R. Posterior Cingulate	0,352	0,73

	TP1-TP2: Difference in change between TR and DR	TP2-TP3: Difference in change between TR and DR
Full model: PBVC ~ group + gender + family risk + handedness<br>Null model: PBVC ~ gender + family risk + handedness		
**ROI**	**BF**	**BF**
L. Entorhinal	0,871	0,70
R. Entorhinal	0,350	0,35
L. Fusiform	0,482	0,37
R. Fusiform	0,903	0,35
L. Inferior Temporal	0,360	0,36
R. Inferior Temporal	0,633	0,38
L. Middle Temporal	0,409	0,46
R. Middle Temporal	0,595	0,37
L. Parahippocampal	0,367	0,37
R. Parahippocampal	0,350	0,36
L. Superior Temporal	0,563	0,82
R. Superior Temporal	0,546	0,42
L. Transverse Temporal	0,400	0,48
R. Transverse Temporal	0,521	0,76
L. Insula	0,687	1,54
R. Insula	0,386	0,37
